# Locked in, Left Behind: Clinical, Psychosocial, and Ethical Dimensions of Prison Health—A Narrative Review

**DOI:** 10.3390/healthcare14193262

**Published:** 2026-10-02

**Authors:** Adelina Ioana Coman, Marius Florentin Popa, Sorin Deacu, Lavinia Simona Neculai-Candea, Miruna Gherase-Cristian, Carmen Corina Radu

**Affiliations:** 1 Doctoral School of Medicine and Pharmacy, George Emil Palade University of Medicine, Pharmacy, Science, 540142 Târgu Mureș, Romania; dr.adelinadcarabulea@gmail.com; 2 Department of Forensic Medicine, County Clinical Emergency Hospital of Constanta, 900591 Constanta, Romania; sorin.deacu@365.univ-ovidius.ro; 3 Faculty of Medicine, Ovidius University, 900470 Constanta, Romania; miruna.cristian@365.univ-ovidius.ro; 4 Center for Research and Development of the Morphological and Genetic Studies of Malignant Pathology-CEDMOG, “Ovidius” University of Constanta, 900470 Constanta, Romania; 5 Department of Forensic Medicine, George Emil Palade University of Medicine, Pharmacy, Science, 540142 Targu Mures, Romania; carmen.radu@umfst.ro; 6 Department of Forensic Medicine, Institute of Forensic Medicine, 540141 Targu Mures, Romania

**Keywords:** prison health, incarcerated populations, correctional healthcare, mental health in prisons, prison overcrowding, bioethics in correctional medicine, infectious diseases in prisons, psychosocial determinants, prisoner rehabilitation, prison systems

## Abstract

**Highlights:**

**What are the main findings?**

- Incarcerated populations carry a disproportionate burden of infectious disease, chronic illness, and mental disorder relative to matched general populations, reflecting the interaction of pre-incarceration socioeconomic and clinical disadvantage with structural conditions of confinement, including prison density above official capacity and constrained healthcare capacity.
- The psychosocial burden of confinement is consistently associated with elevated risk of depression, anxiety, self-harm, and suicide, with risk concentrated at reception and in the period immediately following release.

**What are the implications of the main findings?**

- The reviewed literature indicates that multidisciplinary strengthening of prison healthcare (reception screening, preventive medicine, mental health and substance use treatment, and continuity-of-care planning across the prison–community interface) is the approach most likely to reduce health disparities in this population.
- Aligning prison health policy with international human rights standards is likely to support rehabilitation and reintegration outcomes and to yield broader population health benefits, since most people who are incarcerated eventually return to their communities. These implications are supported by the wider literature; they are not outcomes demonstrated by the present review.

**Abstract:**

The global prison population has expanded over recent decades, straining correctional systems and positioning detention facilities as critical, yet frequently overlooked, sites of health inequity. With an estimated 11.5 million people currently incarcerated worldwide, prisons have become pivotal settings for examining the convergence of clinical vulnerability, psychosocial adversity, and human rights concerns. The aim of this narrative review was to examine how clinical, psychosocial, and bioethical factors interact to shape the health of incarcerated populations and to address four questions: (i) what is the burden and distribution of disease in correctional settings; (ii) which psychosocial consequences of confinement are most consistently reported and through which mechanisms; (iii) what bioethical tensions arise in the delivery of prison healthcare; and (iv) how do these patterns manifest across global, European, and Romanian contexts? We conducted an integrative narrative review reported against the SANRA criteria, searching PubMed, ScienceDirect and Scopus databases, alongside institutional sources from the World Health Organization, the United Nations Office on Drugs and Crime, the Council of Europe, and national correctional authorities, and synthesizing the evidence thematically across the three domains. The evidence indicates that incarcerated populations experience increased rates of infectious disease, chronic illness, mental disorders, and substance use disorders, combined with overcrowding, resource-limited healthcare systems, and restricted access to preventive care. Psychosocial stressors are consistently associated with elevated risk of anxiety, depression, self-harm, and suicide. Ethical tensions persist between institutional security requirements and the obligation to protect the autonomy, dignity, and right to healthcare of people deprived of liberty, equivalent to that available in the community. The contribution of this review is to integrate three literatures that are usually examined separately, the epidemiological, the psychosocial, and the bioethical, into a single conceptual framework spanning three nested levels of analysis and to apply that framework to a Central and Eastern European system that is under-represented in the prison health literature. Because the majority of incarcerated individuals eventually return to their communities, improving prison health is not solely a correctional priority but a public health and social justice imperative; the policy implications set out here follow from the broader literature synthesized in this review rather than from outcomes demonstrated by the review itself.

## 1. Introduction

Deprivation of liberty is among the most severe sanctions available to modern judicial systems. While incarceration serves legitimate societal functions, it also imposes profound and often underappreciated costs on the physical, mental, and social well-being of those confined [1]. Prisons constitute uniquely restrictive social environments in which health determinants, living conditions, and institutional policy converge to shape the trajectories of incarcerated individuals well beyond the period of detention [2,3].

The scale of this challenge is considerable. Approximately 11.5 million people are currently incarcerated worldwide, a figure comprising 10.99 million documented in national statistics and the remainder estimated for jurisdictions that do not report, reflecting decades of punitive sentencing reform, mandatory minimum statutes, and structural inequalities that disproportionately funnel vulnerable populations into the criminal justice system [4].

Three distinct research literatures have converged on prison health, and their intersection provides the analytical structure of this review. First, the clinical and epidemiological literature has established that incarcerated populations carry an excess burden of disease; umbrella reviews of pooled prevalence estimates document elevated rates of psychiatric morbidity, substance use disorders, blood-borne and airborne infections, and chronic non-communicable diseases relative to age- and sex-matched general populations [5,6,7]. Second, the psychosocial literature has shown that this burden cannot be explained by pre-incarceration vulnerability alone: the conditions of confinement themselves are independently associated with psychological distress, self-harm, suicide, and impaired reintegration [8,9,10]. Third, the bioethical and human rights literature has articulated the normative framework governing the response to that burden, centered on the principle of equivalence of care and complicated in practice by dual loyalty, confidentiality constraints, and the limits of consent in a coercive environment [11,12,13]. These three domains are not merely adjacent. The World Health Organization’s health in prisons framework treats the clinical, the social, and the rights-based dimensions as a single system, in which structural determinants generate clinical need, institutional conditions modify it, and normative obligations determine whether it is met. Examining any one domain in isolation therefore risks attributing to individual pathology what is in fact produced by institutional design or attributing to institutional failure what reflects pre-existing social disadvantage. It is this interaction, rather than the three domains taken separately, that the present review sets out to characterize. Table 1 shows the health burden in prison population as it is reported by domain.

Health outcomes among incarcerated individuals are consistently worse than those observed in the general population. Elevated rates of infectious disease, tuberculosis, hepatitis, and HIV co-exist with a substantial chronic disease burden, including cardiovascular disease, diabetes, and respiratory disorders [21]. Mental health disorders, such as depression, anxiety, post-traumatic stress disorder, and substance use disorders, are similarly over-represented in correctional settings [20].

Beyond its clinical burden, incarceration imposes substantial psychosocial costs. The disruption of family and community ties, exposure to pervasive stigma, and the restrictive, often depersonalizing conditions inherent to the carceral environment collectively contribute to psychological distress and the erosion of social identity [10].

The prison system further raises pressing bioethical and human rights concerns (Table 2). International conventions affirm that incarcerated individuals retain fundamental rights, including the right to healthcare, dignity, and humane treatment [11,12,13].

Despite the volume of evidence within each of these three domains, they are rarely integrated. Epidemiological syntheses characterize the burden of disease without systematically situating it within the institutional and normative conditions that produce and sustain it; bioethical analyses articulate obligations without quantifying the need against which those obligations are measured; and the literature remains heavily weighted toward Western European, North American, and Australasian systems, with Central and Eastern European systems comparatively under-examined despite reporting some of the highest prison densities in Europe. The aim of this narrative review is therefore to examine how clinical, psychosocial, and bioethical factors interact to shape the health and well-being of incarcerated populations, integrating evidence across global, European, and Romanian levels of analysis. Four research questions guide the review: (i) What is the burden and distribution of physical and mental disease in correctional settings, and which structural determinants are most consistently implicated? (ii) Which psychosocial consequences of confinement are most consistently reported, and through which mechanisms do they operate? (iii) What bioethical tensions arise in the provision of healthcare to people deprived of liberty, and how are they framed by international human rights instruments? (iv) How do these patterns manifest across global, European, and Romanian contexts, and what do the observed differences imply for policy? By addressing these questions within a single framework, the review aims to provide an integrated account of the structural challenges confronting prison health systems and to specify where multidisciplinary intervention is most likely to be consequential.

## 2. Materials and Methods

### 2.1. Review Design and Rationale

This study was conducted as an integrative narrative review. In this method, literature of heterogeneous design is purposively identified and combined through interpretive rather than aggregative synthesis, with the explicit aim of generating a conceptual account of a complex phenomenon rather than a pooled estimate of effect. Reporting follows the Scale for the Assessment of Narrative Review Articles (SANRA) criteria [32].

This design was deliberately selected over alternatives for three reasons. First, the review question is conceptual and cross-domain: it asks how clinical, psychosocial, and bioethical factors interact, which cannot be expressed as a single PICO-structured question with a defined comparator and outcome. Second, the evidence types are non-commensurable. Prevalence estimates from meta-analyses, findings from qualitative interview studies, judgments of the European Court of Human Rights, and normative instruments such as the European Prison Rules cannot be pooled statistically, nor can they be appraised against a common risk-of-bias instrument; a systematic review with meta-analysis would therefore have required the exclusion of precisely the normative and institutional evidence that the review question requires. Third, a scoping review was considered and rejected: the purpose here is interpretive integration and the construction of an explanatory model, not mapping the volume, distribution, and type of available evidence, which is the characteristic output of a scoping design.

This review was registered in the International Prospective Register of Systematic Reviews (PROSPERO) under registration number CRD420261333995 (full record available in Supplementary Material S5). Four deviations from the originally registered protocol should be noted, each of which has since been corrected in the registration record by published amendment. The original record listed Embase, MEDLINE, PubMed and Scopus as the databases to be searched, whereas the searches conducted used PubMed, ScienceDirect and Scopus. The original record stated that no date restriction would be applied, whereas a primary publication window of 2015 to 2025 was used, with earlier sources retained when foundational. The original record restricted the population to incarcerated adults over 18, whereas this review also considers adolescents held in custodial institutions because the health and developmental consequences of custody in this group are integral to the review question. The original record specified risk-of-bias assessment using Cochrane RoB-2 and ROBIS; neither was applied because neither is applicable to the normative instruments, court judgments and statistical series on which a substantial part of this review depends, and the appraisal approach actually used is described in Section 2.6. These deviations reflect the evolution of the work from a systematic to an integrative narrative design and are disclosed here so that readers can weigh them.

### 2.2. Information Sources and Search Strategy

Three bibliographic databases were searched: PubMed, ScienceDirect and Scopus. These were supplemented by targeted searching of institutional and normative sources that are not indexed in bibliographic databases but are indispensable to the review question: the World Health Organization (including the Regional Office for Europe health in prisons programme), the United Nations Office on Drugs and Crime, the Joint United Nations Programme on HIV/AIDS, the Council of Europe (including the Annual Penal Statistics, SPACE I, and the European Committee for the Prevention of Torture), the World Prison Brief maintained by the Institute for Crime and Justice Policy Research, the HUDOC database of the European Court of Human Rights, the European Union Agency for Fundamental Rights criminal detention database, and the Romanian National Administration of Penitentiaries.

The search strategy combined terms for the custodial setting with terms for health status, healthcare delivery, psychosocial consequences and ethics. The following string was run in PubMed and adapted to the syntax of each remaining database:

(“prison*” OR “prisoner*” OR “incarcerate*” OR “imprison*” OR “detention” OR “detainee*” OR “custodial setting*” OR “correctional facility*” OR “penitentiary”) AND (“health” OR “morbidity” OR “mortality” OR “infectious disease*” OR “tuberculosis” OR “hepatitis” OR “HIV” OR “chronic disease*” OR “mental health” OR “psychiatric disorder*” OR “substance use” OR “suicide” OR “self-harm” OR “healthcare access” OR “health services” OR “equivalence of care” OR “overcrowding” OR “stigma” OR “social isolation” OR “reintegration” OR “ethics” OR “bioethics” OR “human rights” OR “confidentiality” OR “informed consent”)

In ScienceDirect, where the number of Boolean operators per field is restricted, the string was split into four thematic searches covering physical health, mental health, psychosocial consequences and ethics, and the results were combined. In Scopus, the string was applied to title, abstract and keywords using the TITLE-ABS-KEY field. Searches covered material published between January 2015 and December 2025, when the last search was run. Reference lists of the included reviews were checked for additional sources, a technique known as backward citation searching. The institutional and normative sources listed above were not searched by Boolean query, which their platforms do not support; they were consulted directly for the most recent edition of each relevant statistical series, guideline or instrument, and the edition used is identified in the reference list. No unpublished or grey literature was sought beyond these institutional publications. The full search results are included in Supplementary Material S2.

### 2.3. Eligibility Criteria

Table 3 presents the eligibility criteria for inclusion in the study.

### 2.4. Selection of Sources

Records retrieved from the three bibliographic databases were merged, and duplicates were removed. Titles and abstracts were screened independently by two reviewers against, and the full text of every record passing that stage was examined independently by the same two reviewers before a final decision was made (Table S3 in Supplementary Material S3). Disagreements at either stage were resolved by discussion and by referral to a third author where agreement was not reached. Because the review is integrative rather than aggregative, the purpose of screening was to establish relevance and adequacy of reporting rather than to assemble an exhaustive set of studies for pooling, and no attempt was made to identify every publication meeting the criteria. The institutional, statistical and legal sources were selected purposively rather than screened, on the basis that each is the authoritative source for the indicator or norm it reports; where a source issues an annual series, the most recent edition available at the date of the last search was used.

### 2.5. Data Extraction and Charting

**Figure 1:**
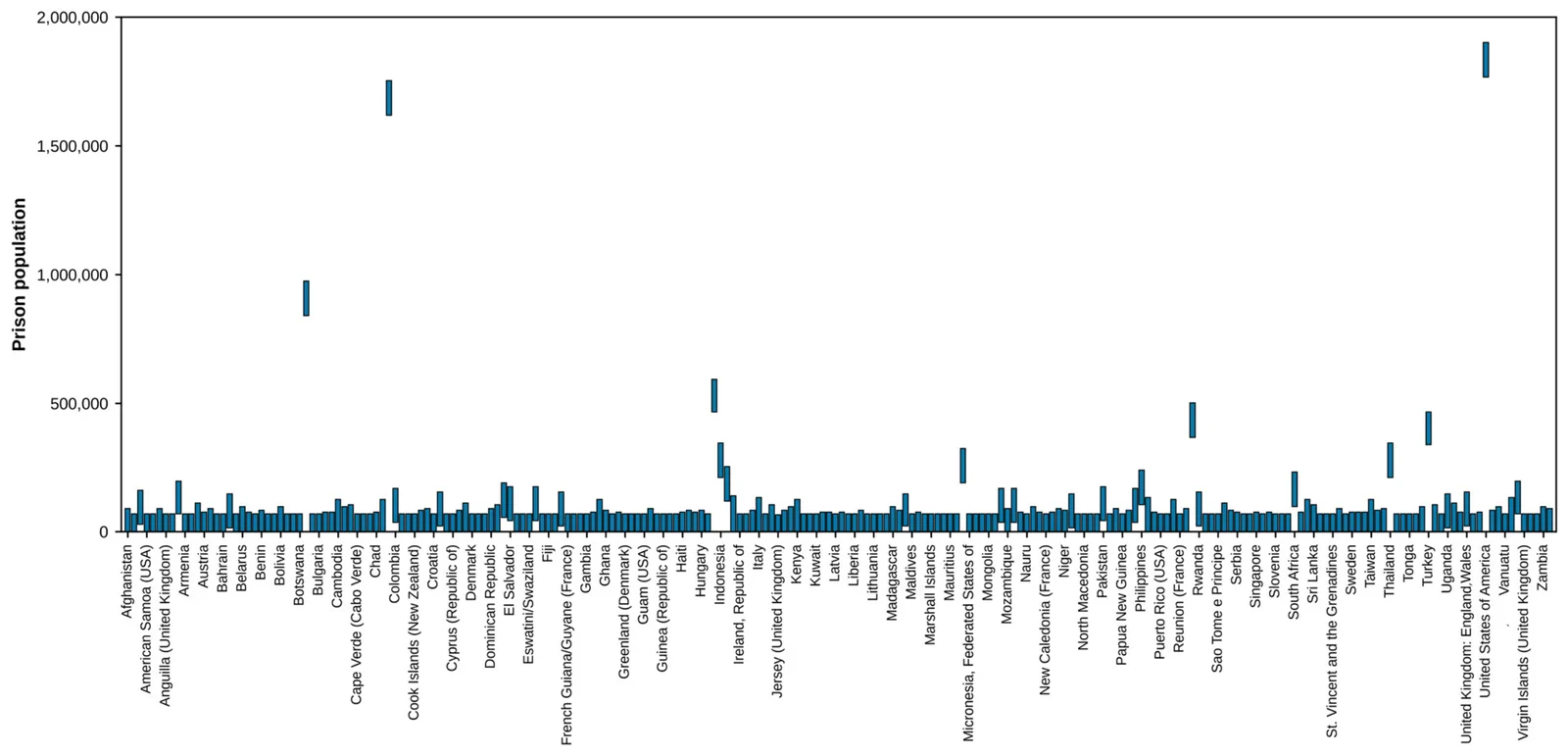
Distribution of total prison population across countries worldwide, 2024. Data source: World Prison Brief, Institute for Crime and Justice Policy Research. Counts are national totals including pre-trial detainees and remand prisoners, recorded at the most recent reference date available for each jurisdiction; reference dates differ between countries, which limits strict comparability. Figure created in IBM SPSS Statistics version 30.0 [5]. Underlying dataset: Supplementary Material S1.

**Figure 2:**
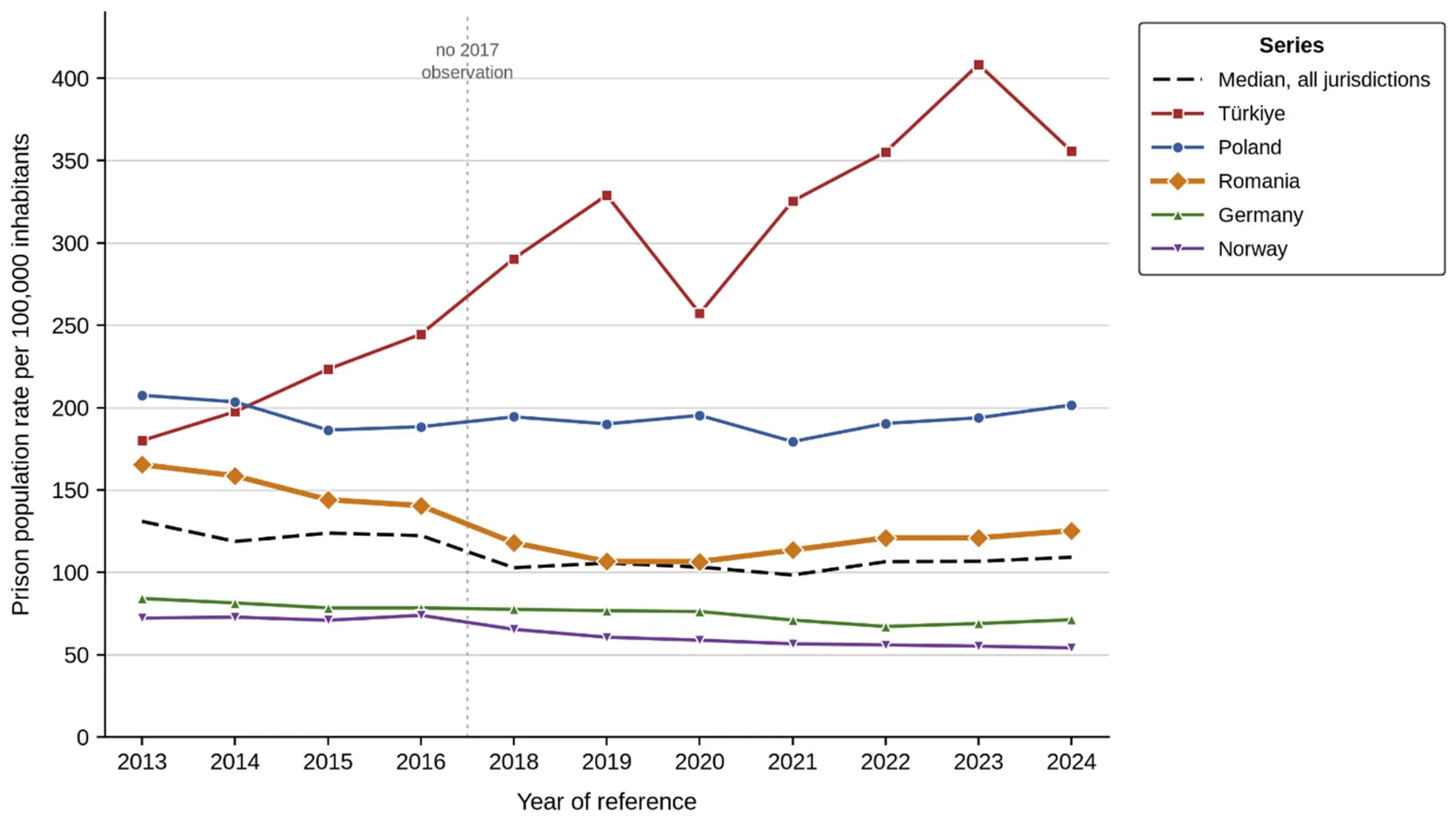
Trends in prison population rates in selected Council of Europe jurisdictions, 2013–2024. Rates are non-adjusted and include pre-trial detainees, expressed per 100,000 inhabitants. The dashed line is the median across all reporting jurisdictions, totaling 48 in 2024, excluding subnational administrations where a national total is also reported. The reference date is 1 September for 2013–2016 and 31 January from 2018 onward; no data are available for 1 September 2017, so the 2016–2018 interval covers 17 months rather than 12, and the horizontal axis is therefore categorical. The underlying series are reproduced in Supplementary Material S3. Data source: Council of Europe Annual Penal Statistics—SPACE I 2024, Table S4 [34].

Data were extracted into a structured matrix organized by the three domains of the review. For empirical sources, the matrix recorded the setting and country, the population and sample size, the design, the health or psychosocial outcome reported, the effect or prevalence estimate with its measure of uncertainty where given, and the authors’ stated limitations. For statistical series, the matrix recorded the indicator, its definition, the reference date, the reporting jurisdictions and any caveat published by the compiling body, since these series are not uniformly defined across countries. For normative instruments and court judgments, the matrix recorded the issuing body, the date, the provision or holding relied upon, and its status as binding or advisory. Extraction was performed by Dr. Sorin Deacu and Dr. Adelina Ioana Coman and checked by Prof. Dr. Marius Florentin Popa. The extracted values underlying Figure 1 and Figure 2 and Table S1 are provided in Supplementary Materials S1, S3 and S4.

### 2.6. Appraisal of the Included Sources

No single appraisal instrument was applied across the evidence base, and we state this explicitly rather than imply a uniform assessment. The sources are not commensurable: a meta-analysis of prevalence, a qualitative interview study, a judgment of the European Court of Human Rights and an annual penal statistics series cannot be scored against a common risk-of-bias tool, and instruments such as Cochrane RoB-2 and ROBIS are not applicable to normative or statistical sources. Each source was instead appraised against criteria appropriate to its type. Systematic reviews and meta-analyses were assessed for the clarity of the review question, the adequacy of the search, whether heterogeneity was quantified and explained, and whether the authors distinguished association from causation. Primary observational studies were assessed for the representativeness of the sample, whether the setting was described sufficiently for the finding to be interpreted, and whether confounding by pre-incarceration vulnerability was addressed. Qualitative studies were assessed for the transparency of sampling and analysis and for the fit between the data presented and the interpretation offered. Statistical series were assessed for the comparability of definitions across jurisdictions, the coverage of reporting, and the presence of published caveats; any value that the compiling body itself flags as unreliable was excluded from the Results. Normative instruments and judgments were treated as statements of standard rather than as evidence of effect and are cited as such.

### 2.7. Synthesis

Synthesis was thematic and interpretive and proceeded in four stages. First, extracted findings were coded inductively within each of the three domains, producing descriptive categories close to the language of the original sources. Second, categories recurring across domains were grouped into candidate analytical themes; the recurrence of a theme across evidence types of different kinds, for example, a prevalence estimate, a qualitative account and a judicial finding converging on the same feature of custody, was treated as the principal criterion of robustness since statistical pooling was not available. Third, the themes were examined for contradiction as well as for convergence, and the points at which sources disagree are reported rather than reconciled. Fourth, the surviving themes were arranged into the five-stage pathway shown later, which links structural and pre-incarceration determinants, prison conditions and institutional constraints, health outcomes, psychosocial consequences, and reintegration. No quantitative pooling, meta-regression or statistical test of heterogeneity was performed, and no pooled estimate is reported anywhere in this review.

## 3. Results

### 3.1. Global Prison Population and Detention Conditions

The global prison population has grown substantially over recent decades. Approximately 11.5 million individuals are currently incarcerated worldwide, of whom 10.99 million are documented in national statistics, with pronounced cross-national variation in incarceration rates and correctional policy [4]. Several countries report exceptionally high incarceration rates, driven by punitive sentencing frameworks, mandatory minimum sentences, and the criminalization of non-violent offenses.

Overcrowding remains among the most consequential structural challenges facing prison systems globally [18]. Many facilities operate well beyond their designed capacity, with direct consequences for living conditions, hygiene, and access to healthcare. There, prisoners frequently contend with inadequate sanitation, insufficient living space, and curtailed opportunities for physical activity and social interaction [19].

### 3.2. Prison Systems in Europe

European prison systems display considerable variation in incarceration policies, detention conditions, and rehabilitation strategies [20]. While some countries emphasize punitive approaches, others have adopted rehabilitation-oriented correctional models.

Recent data from the Council of Europe highlight that prison overcrowding remains a persistent challenge across Europe, affecting approximately one-third of prison administrations. According to the 2024 SPACE I annual penal statistics, the overall number of inmates per 100 available prison places across reporting administrations rose from 93.5 in 2023 to 94.9 in 2024, while the median density stood at 93.6 per 100 places, indicating that the aggregate increase is driven by a subset of severely overcrowded systems rather than by a general tightening of capacity [33,34]. Severe overcrowding was observed in states such as Slovenia, Cyprus, France, Italy, Romania, and Belgium, while several others operated close to maximum capacity, indicating that even minor increases in prison admissions could trigger overcrowding [33,34,35]. Overall, more than one million individuals were detained across Council of Europe member states, with a median incarceration rate of approximately 105 inmates per 100,000 inhabitants. The report emphasizes that overcrowding negatively affects living conditions, limits rehabilitation efforts, and increases institutional pressure on prison systems. Importantly, shorter average detention periods and the wider use of alternative sanctions have been identified as key policy tools for reducing incarceration rates and mitigating prison overcrowding in European correctional systems [33,34,35].

Norway is often cited as an example of a human-centered prison system, focusing on reintegration and maintaining living conditions similar to those in the general community [2]. In contrast, several European countries continue to face challenges related to overcrowding, limited healthcare services, and inadequate infrastructure [35]. Figure 2 illustrates the trends in prison population rates in several countries [34].

The trajectories in Figure 2 diverge in ways that a single cross-sectional rate conceals. The European median fell from 130.9 per 100,000 inhabitants in 2013 to 109.2 in 2024, but this aggregate decline is not reproduced within it. Türkiye rose from 180.0 to 355.7, peaking at 407.8 in 2023, while Norway fell from 72.2 to 54.1 and Germany from 84.1 to 71.2; Poland remained essentially unchanged at approximately 200 throughout. Occupancy relative to capacity varies just as widely and is a distinct measure from the incarceration rate since it depends on the stock of available places as well as on the number of people held. On 31 January 2024, six administrations reported severe overcrowding, with Slovenia at 134.3, Cyprus at 132.2, France at 123.6, Italy at 118.1, Romania at 116.3 and Belgium at 112.7 inmates per 100 places, against a European median of 93.6; Spain, by contrast, reported 74.0 [34]. A country may therefore combine a moderate incarceration rate with acute overcrowding or a high rate with adequate capacity, and the two indicators should not be read interchangeably. European prison regulations emphasize the principle of minimum restriction, meaning that deprivation of liberty should not involve unnecessary limitations beyond those required for security [36]. Additionally, international frameworks require that incarcerated individuals receive healthcare services equivalent to those available in the community [6].

### 3.3. Prison System in Romania

The Romanian penitentiary system has undergone significant transformations since the political transition following the fall of communism [37]. However, structural challenges persist, particularly regarding overcrowding and infrastructure limitations.

Romania ranks 66th among 224 jurisdictions by total prison population (Supplementary Material S1) [3]. On 31 January 2024, the reference date of the SPACE I survey, 23,879 people were held in Romanian penitentiaries, corresponding to a prison population rate of 125.3 per 100,000 inhabitants and to an occupancy of 116.3 inmates per 100 available places, which places Romania among the six Council of Europe administrations reporting severe overcrowding [34]. Despite efforts to expand prison capacity, occupancy continues to exceed available places.

The Romanian prison population rose modestly but consistently across 2024, from 23,879 in January to a peak of 24,699 in November before easing to 24,606 in December, an increase of approximately 3% over the year, with increases recorded in ten of the eleven successive monthly intervals (Supplementary Figure S1 and Table S1, Supplementary Material S4). The absence of any month in which the population fell substantially indicates that the overcrowding documented above reflects a persistent capacity deficit that gradually worsened rather than a transient peak, which is consistent with the 3.6% increase in the Romanian prison population rate between 2023 and 2024 shown in Figure 2. Despite these minor fluctuations, the general pattern suggests a relatively constant prison population, indicating that incarceration levels in Romania during 2024 did not experience major structural changes.

International institutions, including the European Court of Human Rights, have repeatedly highlighted deficiencies in detention conditions within Romanian prisons [38]. These concerns include overcrowding, insufficient healthcare access, poor hygiene conditions, and limited rehabilitation programs.

Nevertheless, recent reforms have aimed to improve prison infrastructure, expand rehabilitation programs, and enhance cooperation with non-governmental organizations to facilitate social reintegration [38,39].

### 3.4. Medical Conditions in Prison Populations

The health burden among incarcerated populations is significantly higher than in the general population. Prisoners commonly experience a combination of communicable diseases, chronic illnesses, and mental health disorders.

Infectious diseases such as tuberculosis, hepatitis B and C, and HIV occur at higher rates due to overcrowding, limited preventive measures, and high-risk behaviors [19,40]. Sexually transmitted infections also present important public health challenges in correctional settings [36].

Chronic conditions including hypertension, diabetes, asthma, and cardiovascular disease require continuous medical monitoring and access to medication, which can be difficult to maintain within resource-constrained prison healthcare systems [41].

Mental health disorders represent one of the most prevalent health issues among incarcerated individuals. Depression, anxiety disorders, and substance use disorders are particularly common. Suicide remains one of the leading causes of death in prisons worldwide [42].

### 3.5. Psychosocial Consequences of Incarceration

The prison environment profoundly affects the psychological well-being of incarcerated individuals. Isolation, loss of autonomy, and limited social interaction contribute to increased levels of stress, anxiety, and depression [22]. Long-term incarceration can lead to reduced self-regulation, cognitive decline, and social withdrawal. Studies suggest that prolonged exposure to restrictive environments may impair psychological functioning and increase the risk of recidivism [29].

Social stigma represents another major challenge faced by incarcerated individuals, particularly after release. Former prisoners frequently encounter discrimination in employment, housing, and social relationships, which complicates their reintegration into society [10]. Supportive social relationships, including family connections and community support, play a critical role in improving mental health outcomes and reducing recidivism rates [24,25].

### 3.6. Ethical Considerations in Prison Healthcare

The provision of healthcare in prisons raises complex ethical questions related to autonomy, confidentiality, and the balance between medical care and institutional security [43].

International human rights frameworks emphasize that prisoners retain their right to healthcare equivalent to that provided in the general community. Healthcare professionals working in correctional settings must therefore maintain professional independence while navigating institutional constraints [44].

Ethical challenges also arise in areas such as informed consent, confidentiality of medical records, management of infectious diseases, and access to specialized treatments. Particular concerns exist regarding vulnerable groups within prison populations, including individuals with mental illness, chronic diseases, and infectious conditions such as HIV [45].

## 4. Discussion

### 4.1. What the Evidence Consistently Shows

Four findings recur across the literature with sufficient consistency to be treated as established. First, the excess burden of disease in prison populations is large, multi-domain, and reproduced across health systems of widely differing resource levels; it is not an artefact of any single national system [46,47]. Second, that burden is concentrated at the two ends of the custodial trajectory: need is already elevated at reception, reflecting the socioeconomic and clinical characteristics of those who enter custody, and risk rises sharply again in the weeks immediately following release [36,41]. Third, structural conditions (prison density above official capacity, constrained clinical staffing, and discontinuity of care at transfer and release) are consistently identified as proximal determinants of poorer outcomes across studies using very different designs [34]. Fourth, the normative framework is unusually settled while its implementation is not: the principle of equivalence of care is affirmed in binding and non-binding instruments alike and is largely uncontested in the literature, yet the same literature documents its systematic non-attainment. The interpretive value of these four findings lies in their conjunction [48]. They indicate that prison health outcomes are produced neither solely by the characteristics of the people who are incarcerated nor solely by the institutions that hold them but by the interaction of imported vulnerability with institutional capacity, under a normative regime that specifies the obligation without securing the resources for its discharge.

### 4.2. Inconsistencies, Heterogeneity, and Contested Findings

**Figure 3:**
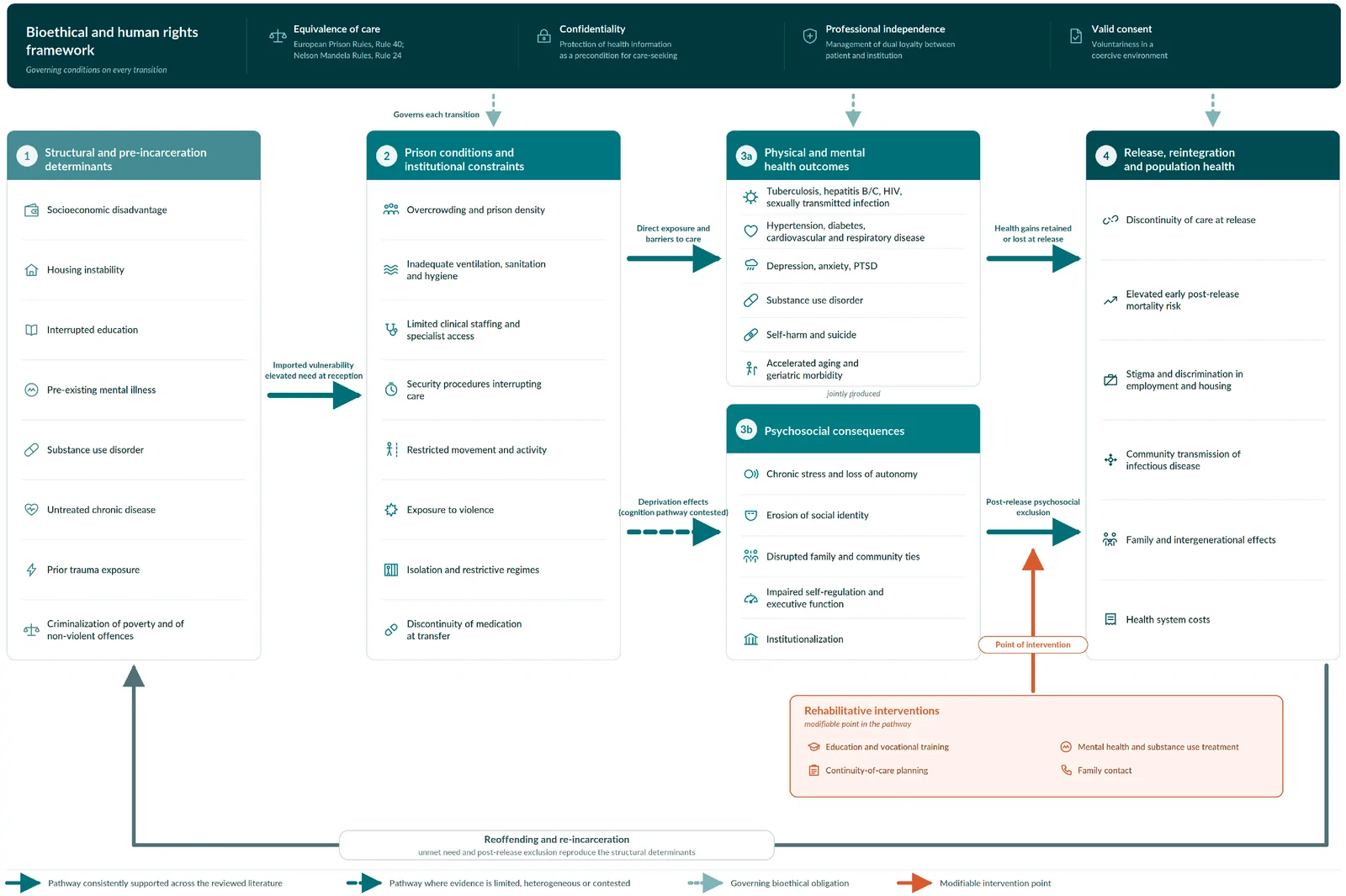
Conceptual model of the pathways linking structural determinants, prison conditions, health outcomes, psychosocial consequences, and reintegration. The model integrates the three domains examined in this review. Structural and pre-incarceration determinants (left) generate clinical and psychosocial need that is already elevated at the point of reception. Prison conditions and institutional constraints (center-left) modify that need, acting as both direct exposures and barriers to care. Physical and mental health outcomes and psychosocial consequences (center-right) are therefore jointly produced by imported vulnerability and by the environment of confinement, a distinction that available evidence does not fully resolve. Release and reintegration (right) determine whether health gains achieved in custody are retained, with post-release stigma, discontinuity of care, and housing and employment exclusion feeding back into reoffending and re-incarceration (lower feedback pathway). Bioethical obligations (equivalence of care, confidentiality, professional independence, and valid consent) operate as governing conditions on each transition rather than as a separate stage (upper band).

Several areas central to this review remain genuinely contested, and the strength of the conclusions that can be drawn differs accordingly. The most consequential concerns the relative contribution of pre-incarceration vulnerability, or importation, versus the effects of confinement itself, or deprivation [49,50]. Reception cohort studies show that a substantial proportion of psychiatric and substance use morbidity is present on entry, while longitudinal and qualitative evidence shows deterioration during custody; few studies are designed to separate the two, and the absence of appropriate community comparison groups matched on socioeconomic exposure means that the attributable fraction remains unresolved [51]. A second area concerns the cognitive effects of restrictive confinement, where findings are heterogeneous, sample sizes are typically small, exposure definitions vary between jurisdictions, and the possibility of selection into restrictive regimes on the basis of pre-existing impairment is rarely addressed; we have therefore drawn this pathway as provisional in Figure 3.

A third concerns cross-national comparison of recidivism, where apparent differences between punitive and rehabilitative systems are confounded by differing definitions of reoffending, differing follow-up periods, differing thresholds for prosecution, and differing composition of the prison population; system-level comparisons of this kind should be read as generating hypotheses rather than as estimating policy effects [52,53]. A fourth concerns pandemic-era decarceration, where reductions in prison density were accompanied by intensified isolation and suspension of visits; thus, the net health effect reported in the literature is mixed [54,55].

### 4.3. Mechanisms Linking Incarceration to Health

Five mechanisms recur across the reviewed literature and are represented as the principal pathways in Figure 3. The first is concentration: custody assembles, in a confined space, a population selected for high prevalence of transmissible infection, untreated chronic disease, and psychiatric and substance use morbidity, which raises baseline need independently of anything the institution does. The second is environmental exposure: density above capacity, restricted ventilation, and constrained hygiene convert that concentration into transmission, most clearly for airborne and blood-borne infection. The third is interruption: security procedures, transfers between facilities, and the discontinuity between prison and community health services repeatedly break treatment continuity, which is particularly consequential for conditions requiring uninterrupted pharmacotherapy (antiretroviral, antitubercular, antipsychotic, and opioid agonist treatment alike). The fourth is psychosocial deprivation: loss of autonomy, restricted social contact, and exposure to threat sustain chronic stress responses and erode the social ties that protect against depression and self-harm. The fifth is institutional dependency and stigma, operating principally after release: the same characteristics that facilitate adaptation to a highly regulated environment impede reintegration into an unregulated one, while a criminal record restricts access to the employment, housing, and social relationships on which post-release health depends. These mechanisms are not independent; they compound.

### 4.4. Implications for Clinical Practice and Policy

Clinically, the concentration of need at reception and at release identifies both points as the highest-yield targets for intervention: systematic screening at reception for infection, chronic disease, psychiatric morbidity, and substance use, and structured continuity-of-care planning before release, including medication supply, appointment scheduling, and registration with community services [56]. At the level of the institution, the recurrence of prison density as a determinant across all three domains implies that measures reducing density function as health interventions and not solely as administrative ones; the same applies to protecting clinical contact time from security-related interruption and to safeguarding the confidentiality and professional independence of prison healthcare staff, which the reviewed literature identifies as preconditions for care-seeking rather than as procedural formalities [45]. At the level of policy, because the great majority of people who are incarcerated return to their communities, prison health outcomes are community health outcomes; the reviewed evidence therefore supports treating correctional health services as an integral component of national public health systems, with commissioning, data, and quality standards aligned accordingly, rather than as a separate correctional function.

### 4.5. Limitations

Several limitations should be considered when interpreting this review. First, the design is narrative and interpretive rather than systematic. Second, the review is susceptible to selection and publication bias. Restriction to English-language publications, apart from a small number of Romanian institutional and legal sources, will have excluded relevant evidence, particularly from Latin America, francophone and lusophone Africa, and parts of Asia. Because studies reporting elevated morbidity in custody are more likely to be published than null findings, the pooled picture presented in the literature may overstate the consistency of the association. Third, the evidence base is heterogeneous in ways that constrain generalization. Prison systems differ in size, regime, sentencing framework, and the organization and funding of healthcare, and studies differ in whether populations were sampled at reception, during custody, or after release, which systematically affects reported prevalence; findings should therefore not be transferred between jurisdictions without explicit consideration of system context. Also, no formal risk-of-bias assessment or quantitative pooling was undertaken, and no pooled estimates are reported; all prevalence figures are those published in the cited sources and inherit their limitations. At the same time, the Romanian material draws on national administrative and institutional data of differing granularity and periodicity, which limits the precision of comparison with other European systems. Finally, the conceptual model in Figure 3 is an organizing framework derived from the synthesis; it has not been empirically tested, and the relative magnitude of the pathways it represents remains unquantified.

## 5. Conclusions

The prison environment represents a complex intersection of clinical, psychosocial, and ethical challenges. The literature synthesized in this review indicates that incarcerated populations experience disproportionately high rates of physical illness, mental disorder, and social marginalization and that this burden is produced jointly by pre-incarceration disadvantage, by the structural conditions of confinement, and by the incomplete realization of the principle of equivalence of care.

Improving prison health systems requires coordinated action by healthcare professionals, policymakers, correctional institutions, and community organizations. On the basis of the evidence reviewed, the priorities most consistently supported are systematic health assessment at reception, protection of clinical continuity during custody and across the prison–community interface, reduced prison density, expansion of mental health and substance use treatment, and safeguarding confidentiality and professional independence in correctional healthcare.

Addressing these issues matters both for the rights and dignity of people deprived of liberty and for population health since the great majority of those held in custody return to their communities. It should be emphasized, however, that the potential benefits identified here are implications derived from the broader literature synthesized in this review, not outcomes demonstrated by the review itself. What the present synthesis does support is the conclusion that prison health is inseparable from public health and that a multidisciplinary approach integrating clinical care, psychosocial support, and adherence to international human rights standards offers the most coherent framework for improvement.

## Figures and Tables

**Table 1:** Reported health burden in prison populations by domain.

Domain	Key Reported Findings and Associations	Supporting Evidence
Infectious disease	Tuberculosis, hepatitis B and C, and HIV are reported at substantially higher prevalence than in matched general populations; transmission risk is associated with high prison density, inadequate ventilation, delayed diagnosis, and interrupted treatment on transfer or release. Sexually transmitted infection is also over-represented.	Umbrella review of prison morbidity [6]; WHO Regional Office for Europe report on infectious disease in prison settings; systematic review of respiratory transmission models in carceral settings [14]; UNAIDS.
Chronic non-communicable disease	Hypertension, diabetes, asthma, and cardiovascular disease require continuous monitoring and uninterrupted medication supply; poorer control is associated with limited clinical staffing, security-related interruption of appointments, and discontinuity of care at transfer and release. Burden is amplified by accelerated aging of prison populations.	Umbrella review of prison morbidity [6]; review of long-term condition management in prisons [15]; the literature on incarceration and health in older adults [16].
Mental disorders	Depression, anxiety, and post-traumatic stress disorder are consistently over-represented; meta-analytic estimates of severe mental illness across 43 countries confirm elevated prevalence relative to community samples. Both pre-incarceration vulnerability and conditions of confinement are implicated.	Meta-analysis of severe mental illness across 43 countries [17]; review of prisoner mental health [7].
Substance use disorders and comorbidity	Alcohol and opioid use disorders are highly prevalent at reception; comorbidity of serious mental illness with substance use disorder is reported at rates well above community estimates and is associated with poorer treatment engagement and higher post-release mortality risk.	Meta-analysis of comorbid serious mental illness and substance use disorders in prison populations [8]; analysis of the structure of comorbidity in prison populations [18].
Suicide and self-harm	Suicide is among the leading causes of death in custody, with rates consistently exceeding those of the general population; identified risk factors include recent reception, single-cell occupancy, remand status, and prior self-harm.	Systematic review and meta-analysis of risk factors for suicide in prisons [9]; meta-regression of worldwide prison suicide incidence [19]; epidemiological study of self-harm in prisons in England and Wales [20].

**Table 2:** Psychosocial dimensions of incarceration: reported findings and evidence.

Domain	Key Reported Findings and Associations	Supporting Evidence
Social isolation and restricted contact	Reduced contact with family and community is associated with higher levels of psychological distress, depressive symptoms, and self-harm; maintained family contact during custody is associated with better mental health and lower post-release reoffending.	Psychological impact of incarceration [22]; qualitative study of psychological stressors and coping among older incarcerated persons [23]; studies of family contact during incarceration [24,25].
Loss of autonomy and institutional control	Restricted decision-making, limited privacy, and regimented routine are associated with chronic stress responses and reduced self-efficacy; effects are reported to be more pronounced with longer duration of confinement and in more restrictive regimes.	Psychological impact of incarceration [22]; cluster-randomized trial of cognitive decline and intervention in custody [26].
Restricted environmental stimulation and cognition	Limited educational, occupational, and social stimulation is associated with poorer performance on measures of executive function and self-regulation; the direction of effect is difficult to separate from pre-existing differences, and evidence remains limited and heterogeneous.	Cluster-randomized trial in custodial settings [26]; systematic review and meta-analysis of the health of older offenders [27].
Stigma and post-release exclusion	A criminal record is associated with reduced access to employment, housing, and stable social relationships after release; housing instability in particular is reported as a mechanism linking stigma to re-incarceration.	Study of stigma, housing, and identity after prison [10].
Access to education and programs	Participation in correctional education and vocational programs is associated with lower reoffending in meta-analysis; effect sizes vary by program type, intensity, and evaluation design.	RAND meta-analysis of correctional education and recidivism [28]; research synthesis on treating offenders with mental illness [29].
Cross-cultural and demographic variation	Psychological distress and coping strategies vary by cultural background, age, and sex; findings from one national system do not transfer directly to another.	Study of distress and coping in cross-cultural prison populations [30]; qualitative research on aging and health during and after incarceration [31].

**Table 3:** Eligibility Criteria.

Criterion	Included	Excluded
Population	Adults and adolescents held in prisons, jails, remand facilities, or other closed correctional settings; formerly incarcerated individuals in the post-release period; correctional healthcare staff.	Populations in immigration detention, psychiatric forensic hospitals without a custodial sentence, and military detention, except when reported jointly with prison populations.
Topic	Physical or mental health status, healthcare delivery, psychosocial consequences of confinement, rehabilitation and reintegration, and the ethical or legal framework governing prison healthcare.	Criminological studies of offending behavior, sentencing policy, or prison management without a stated health dimension.
Publication type	Systematic reviews, meta-analyses, umbrella reviews, primary observational and interventional studies, qualitative studies, ethical and legal analyses and reports, statistical series, guidelines, and legal instruments issued by international organizations, national correctional authorities, or courts.	Conference abstracts without full text, editorials and commentaries without original argument, student theses, and preprints that had not undergone peer review at the time of the final search.
Language	English, with Romanian-language institutional and legal sources included where they provided national data not otherwise available.	All other languages.
Time window	Primary window 2015–2025. Sources published before 2015 were included only where they are foundational to the concept under discussion, such as the European Prison Rules, or where no more recent equivalent exists.	Superseded editions of guidelines and statistical series where a current edition existed.

## Data Availability

No new primary data were generated in this study. All data analyzed are contained in this article and its four Supplementary Materials. The data were compiled from the World Prison Brief (https://www.prisonstudies.org), the Council of Europe Annual Penal Statistics SPACE I 2024, and the monthly statistical bulletins of the Romanian National Administration of Penitentiaries (https://anp.gov.ro (accessed on 16 June 2026)), all accessed on 11 July 2026. The review protocol is registered in PROSPERO under CRD420261333995.

## Supplementary Materials

The following supporting information can be downloaded at: https://www.mdpi.com/article/10.3390/healthcare14193262/s1, Supplementary Material S1: country-level prison population totals and world ranking for 224 jurisdictions, compiled from the World Prison Brief. Supplementary Material S2: search results. Supplementary Material S3: the source data underlying Figure 2, comprising SPACE I 2024 Table S2 on special categories of inmates included in national totals, Table S3 on adjusted and non-adjusted prison population rates on 31 January 2024, and Table S4 on trends in prison population rates from 2013 to 2024, each with accompanying notes on variable definitions and data caveats. Supplementary Material S4: Table S1 and Figure S1, the monthly number of people held in Romanian penitentiaries during 2024. Supplementary Material S5: PROSPERO registration record.
