# Supplementary material for: Locked in, Left Behind: Clinical, Psychosocial, and Ethical Dimensions of Prison Health—A Narrative Review
Source: Healthcare (Basel). 2026 Oct 2;14(19):3262. doi: 10.3390/healthcare14193262 (PMC13665056; doi:10.3390/healthcare14193262)
Supplement: Supplementary file 1 [file healthcare-14-03262-s001.zip › Supplementary material S5.pdf]

NIHR | National Institute for Health and Care Research

PROSPERO
International prospective register of systematic reviews

Locked In, Left Behind: Clinical, Psychosocial, and Ethical Dimensions of Prison Health. A Narrative Review

Adelina Ioana Carabulea, Marius Florentin Popa, Sorin Deacu, Carmen Corina Radu

REVIEW TITLE AND BASIC DETAILS

Review title

Locked In, Left Behind: Clinical, Psychosocial, and Ethical Dimensions of Prison Health. A Narrative Review

Condition or domain being studied

The review focuses on the health status, psychosocial determinants, and bioethical challenges affecting incarcerated populations. It examines infectious diseases, chronic conditions, and mental health disorders in prison settings, as well as the structural and psychosocial factors influencing health outcomes and access to healthcare among prisoners.

Rationale for the review

Incarcerated populations represent a highly vulnerable group with a disproportionately high burden of infectious diseases, chronic illnesses, and mental health disorders compared with the general population. Structural determinants such as overcrowding, limited access to healthcare services, socioeconomic vulnerability, and psychosocial stressors associated with incarceration contribute to significant health inequalities within prison systems worldwide. Although numerous studies have examined individual aspects of prison health, including infectious diseases, mental health, or prison conditions, the available evidence is often fragmented and rarely integrates clinical, psychosocial, and bioethical perspectives. This review aims to synthesize the existing literature on the health status, psychosocial determinants, and ethical challenges affecting incarcerated individuals.

Review objectives

The objective of this review is to synthesize existing evidence regarding the health status, psychosocial determinants, and bioethical challenges affecting incarcerated populations. Specifically, the review aims to examine the prevalence and types of physical and mental health conditions in prison populations, including infectious diseases, chronic illnesses, and mental health disorders. In addition, the review seeks to explore the psychosocial factors associated with incarceration, such as social isolation, stigma, and limited autonomy, and their impact on health outcomes. Finally, the review aims to analyze ethical considerations related to healthcare delivery in correctional settings, including issues of access to care, human rights, and equivalence of healthcare for incarcerated individuals.

Keywords

Prison health; Incarcerated populations; correctional healthcare; mental health in prisons; prison overcrowding; bioethics in correctional medicine; infectious diseases in prisons; Psychosocial determinants; prisoner rehabilitation; prison systems

Country

Romania

ELIGIBILITY CRITERIA

Population 1 change

Included

People held in prisons, jails, remand facilities and other custodial institutions, comprising both incarcerated adults aged 18 years and over and adolescents held in custodial institutions. The original registration restricted the population to adults over 18; adolescents in custody were subsequently included because the health and developmental consequences of custody in this group are integral to the review question and cannot be separated from it.

Excluded

People under community supervision, on probation or on parole without a period of custody, and community-based offender populations where the exposure is not detention itself.

Intervention(s) or exposure(s)

Included
In this review, the primary exposure of interest is incarceration and the prison environment. Studies will be included if they examine health outcomes, psychosocial determinants, or ethical issues associated with detention or imprisonment. Relevant exposures may include prison conditions such as overcrowding, limited healthcare access, social isolation, institutional policies, and structural determinants affecting incarcerated individuals. Studies evaluating the impact of incarceration on physical health, mental health, infectious diseases, chronic diseases, psychosocial well-being, or healthcare access among prisoners will be considered eligible.

Excluded
Studies will be excluded if they do not specifically examine incarcerated populations or prison environments as the primary exposure. Research focusing exclusively on the general population, probation populations, or community-based offenders without direct relevance to incarceration will not be included. Studies that do not address health outcomes, psychosocial determinants, or ethical aspects related to prison settings will also be excluded.

Comparator(s) or control(s)
This review does not have any comparators

Study design
Both randomized and nonrandomized study types will be included.

Included
Observational and descriptive studies examining health conditions, psychosocial determinants, or ethical aspects related to incarcerated populations will be included. Eligible study designs may include cross-sectional studies, cohort studies, case–control studies, epidemiological studies, and population-based studies conducted in prison settings. Qualitative studies addressing psychosocial experiences, prison conditions, or ethical issues related to healthcare delivery in correctional environments will also be considered. In addition, relevant reviews, institutional reports, and public health reports providing data on prison health, prison conditions, or correctional healthcare systems may be included to provide contextual information

Excluded
Studies that do not focus on incarcerated populations or prison environments will be excluded. Editorials, opinion papers without empirical or documented evidence, conference abstracts without sufficient methodological information, and studies unrelated to health, psychosocial determinants, or ethical aspects of incarceration will not be included. Animal studies and purely experimental laboratory research not involving prison populations will also be excluded

Context
The review focuses on studies conducted in correctional settings, including prisons, detention centers, and other custodial institutions where individuals are deprived of liberty. The context includes healthcare systems operating within prison environments and the structural conditions that influence the health and well-being of incarcerated populations. Studies from any geographical region will be considered, including high-, middle-, and low-income countries. The review will examine research addressing physical health conditions, mental health disorders, psychosocial determinants, prison conditions, and ethical issues related to healthcare delivery in correctional systems. Both institutional and public health perspectives will be considered in order to capture the complex interaction between prison environments, healthcare access, and prisoner well-being.

TIMELINE OF THE REVIEW

Date of first submission to PROSPERO
06 March 2026

Review timeline
Start date: 6 March 2026. End date: 7 April 2026.

Date of registration in PROSPERO
09 July 2026

AVAILABILITY OF FULL PROTOCOL

Availability of full protocol

A full protocol has been written and uploaded to PROSPERO. The protocol may be accessed through this link https://www.crd.york.ac.uk/PROSPEROFILES/84caf8a9fbaf8e3bef3fbd79236948b0.pdf.

SEARCHING AND SCREENING

Search for unpublished studies
Only published studies will be sought.

Main sources that will be searched 1 change
The main sources to be searched are *PubMed* and *Scopus*.

Other sources that will be searched
ScienceDirect

Search language restrictions
The review will only include studies published in English and Romanian.

Search date restrictions 1 change
Databases will be searched for articles published from 1 January 2015 and before by 31 December 2025.

Other methods of identifying studies 1 change
Other studies will be identified by: *reference list checking (backward citation searching)*.

Link to search strategy 1 change
A full search strategy has been uploaded to PROSPERO. The PDF may be accessed through this link https://www.crd.york.ac.uk/PROSPEROFILES/20e57480178bbe3f2639a69e2fa084b0.pdf.

Selection process
Studies will be screened independently by at least two people (or person/machine combination) with a process to resolve differences.

Other relevant information about searching and screening
None

DATA COLLECTION PROCESS

Data extraction from published articles and reports 1 change
Data will be extracted independently by at least two people (or person/machine combination) with a process to resolve differences.

Authors will not be contacted for further information.

Study risk of bias or quality assessment 1 change
Risk of bias will be assessed using:

No single risk-of-bias tool will be applied. Each source will be assessed using criteria appropriate to its design, considering sampling, representativeness, confounding, transparency, reporting quality, heterogeneity, and methodological limitations. Normative and statistical sources will be evaluated for reliability, comparability, and reporting completeness.

Data will be assessed independently by at least two people (or person/machine combination) with a process to resolve differences.

Additional information will **not** be sought from study investigators if required information is unclear or unavailable in the study publications/reports.

Reporting bias assessment
Potential reporting bias will be assessed by comparing outcomes reported across studies and identifying selective outcome reporting or missing data. Where possible, study protocols and registered reports will be consulted to detect discrepancies between planned and reported outcomes.

Certainty assessment
Certainty of findings will not be assessed

OUTCOMES TO BE ANALYSED

Main outcomes

The main outcomes include the prevalence and types of physical and mental health conditions among incarcerated populations, including infectious diseases (e.g., tuberculosis, HIV, viral hepatitis), chronic diseases (e.g., cardiovascular disease, diabetes, respiratory disorders), and mental health disorders (e.g., depression, anxiety, substance use disorders). Additional outcomes include psychosocial determinants affecting prisoner well-being, such as social isolation, stigma, and loss of autonomy. The review will also examine ethical and healthcare-related outcomes, including access to medical services, quality of healthcare delivery in correctional settings, and adherence to the principle of equivalence of care between prison healthcare systems and community healthcare services

Additional outcomes

Additional outcomes may include prison environmental factors influencing health, such as overcrowding, hygiene conditions, and availability of healthcare resources. Other outcomes include access to preventive services, vaccination coverage, screening programs, rehabilitation initiatives, and health education programs within prisons. The review may also examine reintegration-related outcomes, including post-release health challenges, continuity of healthcare after incarceration, and the impact of prison health policies on public health and social reintegration.

PLANNED DATA SYNTHESIS

Strategy for data synthesis

Data will be synthesized using a narrative and thematic synthesis approach. Findings from eligible studies will be grouped according to major thematic domains, including physical health conditions, mental health outcomes, psychosocial determinants, and ethical aspects of healthcare in prison settings. Studies will be compared based on population characteristics, study design, and prison context. Where appropriate, similarities and differences across studies will be identified to highlight patterns, trends, and gaps in the literature. Quantitative findings will be summarized descriptively, while qualitative findings will be integrated to provide contextual interpretation of the health and social factors affecting incarcerated populations.

CURRENT REVIEW STAGE

Stage of the review at this submission

Review stage	Started	Completed
Pilot work	✓	✓
Formal searching/study identification	✓	✓
Screening search results against inclusion criteria	✓	✓
Data extraction or receipt of IPD	✓	✓
Risk of bias/quality assessment	✓	✓
Data synthesis	✓	✓

Review status

The review is completed.

Publication of review results

Results of the review will be published in English.

REVIEW AFFILIATION, FUNDING AND PEER REVIEW

Review team members 1 change

Adelina Ioana Carabulea (review guarantor and contact) UNIVERSITATEA DE MEDICINĂ, FARMACIE, ŞTIINŢE ŞI TEHNOLOGIE „GEORGE EMIL PALADE" DIN TÎRGU MUREŞ. Romania.

No conflict of interest declared.

Professor Marius Florentin Popa. adelina.marinescu@umft.ro (A.R.M.) 5 Department of Forensic Medicine, "Sf. Apostol Andrei" Emergency County Hospital, 900439 Constanta, Romania. Romania.

No conflict of interest declared.

Dr Sorin Deacu. Department of Forensic Medicine, "Sf. Apostol Andrei" Emergency County Hospital, 900439 Constanta, Romania. Romania.

No conflict of interest declared.

Carmen Corina Radu. Doctoral School of Medicine and Pharmacy, George Emil Palade University of Medicine, Pharmacy, Science and Technology of Târgu Mureș, Romania. Romania.

No conflict of interest declared.

Named contact

Adelina Ioana Carabulea (dr.adelinadcarabulea@gmail.com). UNIVERSITATEA DE MEDICINĂ, FARMACIE, ŞTIINŢE ŞI TEHNOLOGIE „GEORGE EMIL PALADE" DIN TÎRGU MUREŞ. Romania.

Review affiliation 1 change

Universitatea de medicină, farmacie, ştiinţe şi tehnologie

„George Emil Palade" din Targu Mureş

Funding source

Review has no funding and no agreed support from an academic institution and is done in authors' own time.

Peer review

There has been no peer review of this planned review.

ADDITIONAL INFORMATION

Additional information

This review aims to provide an integrated perspective on prison health by combining clinical, psychosocial, and ethical dimensions affecting incarcerated populations. The findings may contribute to improving understanding of health inequalities in correctional settings and support the development of policies and interventions aimed at strengthening prison healthcare systems and promoting prisoner well-being.

Review conflict of interest

Declared individual interests are recorded under team member details.. No additional interests are recorded for this review.

Medical Subject Headings

Chronic Disease; Communicable Diseases; Health Services Accessibility; Health Status; Human Rights; Humans; Incarceration; Mental Health; Organizational Policy; Outcome Assessment, Health Care; Prevalence; Prisoners; Prisons; Social Isolation

Revision note

This revision aligns the record with the review as conducted and reported. The bibliographic databases searched are corrected to PubMed, ScienceDirect and Scopus; Embase.com and MEDLINE were not searched. A publication window of 2015 to 2025 is recorded, replacing the original statement of no date restriction. The population is broadened to include adolescents held in custodial institutions alongside incarcerated adults. The risk-of-bias field is corrected: Cochrane RoB-2 and ROBIS were not applied and are not applicable to the normative instruments, court judgments and statistical series that form part of the evidence base, and the type-specific appraisal actually used is described instead. Related fields on reporting bias, contacting study authors and other methods of identifying studies are corrected for consistency, the search strategy is now supplied, the stage table is completed to record that formal searching was carried out, the review status is updated to complete, and the review team is extended to the full author list of the resulting manuscript. These changes reflect the evolution of the work from a systematic to an integrative narrative design and are disclosed as deviations from the registered protocol in the manuscript itself.

SIMILAR REVIEWS

Check for similar records already in PROSPERO

PROSPERO identified a number of existing PROSPERO records that were similar to this one (last check made on 6 March 2026). These are shown below along with the reasons given by that the review team for the reviews being different and/or proceeding.

- Prevalence and Health Outcomes of Asthma in Incarcerated Populations: A Systematic Review and Meta-Analysis [published 21 September 2024] [CRD42024588971]. The review was judged **not to be similar**
- Facilitators and Barriers Affecting Access to Mental Health Services in Nepal: A Systematic Review [published 18 February 2026] [CRD420261320390]. The review was judged **not to be similar**

- Psychosocial and mental health impacts on children and adults in Ukraine: a systematic review [published 5 February 2025] [CRD42025641830]. The review was judged **not to be similar**

PROSPERO version history 1 change

- Version 3.0, published 19 Sep 2026
- Version 2.1, published 19 Sep 2026
- Version 2.0, published 05 Aug 2026
- Version 1.2, published 05 Aug 2026
- Version 1.1, published 05 Aug 2026
- Version 1.0, published 09 Jul 2026

Disclaimer

The content of this record displays the information provided by the review team. PROSPERO does not peer review registration records or endorse their content.

PROSPERO accepts and posts the information provided in good faith; responsibility for record content rests with the review team. The guarantor for this record has affirmed that the information provided is truthful and that they understand that deliberate provision of inaccurate information may be construed as scientific misconduct.

PROSPERO does not accept any liability for the content provided in this record or for its use. Readers use the information provided in this record at their own risk.

Any enquiries about the record should be referred to the named review contact
