# Supplementary material for: Locked in, Left Behind: Clinical, Psychosocial, and Ethical Dimensions of Prison Health—A Narrative Review
Source: Healthcare (Basel). 2026 Oct 2;14(19):3262. doi: 10.3390/healthcare14193262 (PMC13665056; doi:10.3390/healthcare14193262)
Supplement: Supplementary file 1 [file healthcare-14-03262-s001.zip › Supplementary material S4.pdf]

SUPPLEMENTARY MATERIAL 4

Table S1: Number of people held in Romanian penitentiaries during 2024.

| Month | Number of Prisoners |
|---|---|
| January 2024 | 23879 |
| February 2024 | 23848 |
| March 2024 | 24133 |
| April 2024 | 24300 |
| May 2024 | 24484 |
| June 2024 | 24520 |
| July 2024 | 24602 |
| August 2024 | 24686 |
| September 2024 | 24652 |
| October 2024 | 24651 |
| November 2024 | 24699 |
| December 2024 | 24606 |

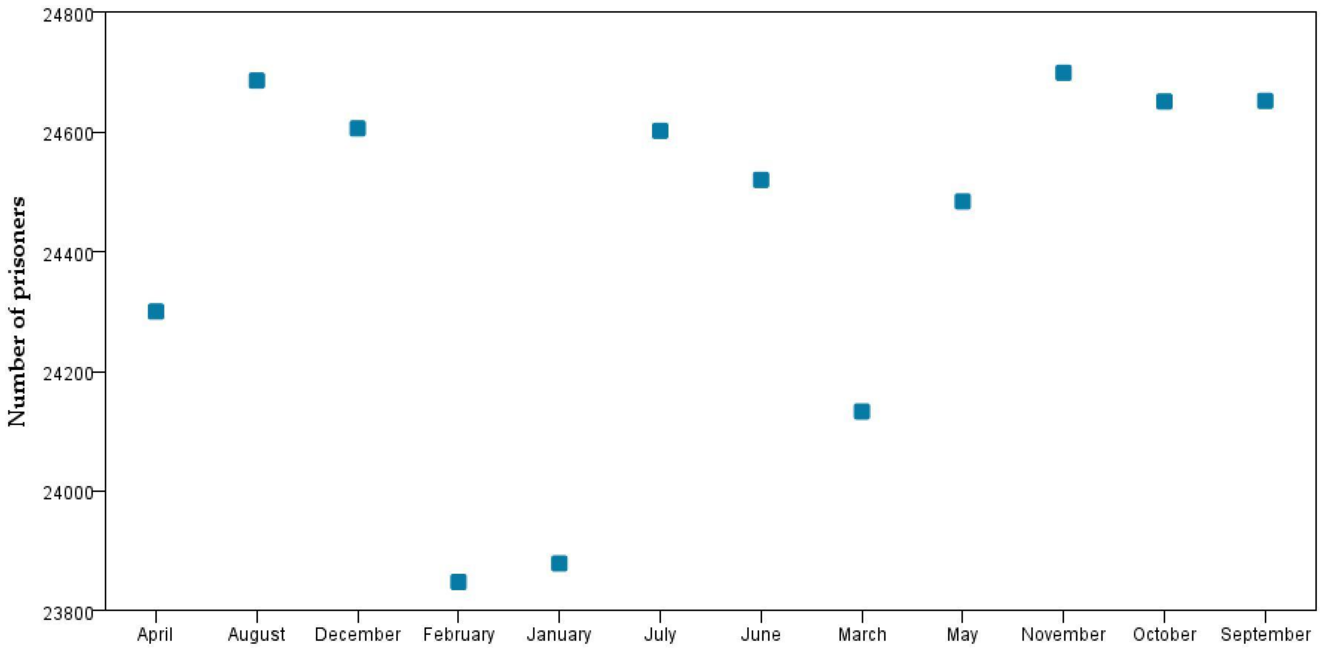


Supplementary Figure S1. Monthly variation in the number of people held in Romanian penitentiaries during 2024. Data source: monthly statistical bulletins of the National Administration of Penitentiaries, Romania. Figure created in IBM SPSS Statistics version 30.0
